# A low-calorie meal replacement improves body composition and metabolic parameters in shift workers with overweight and obesity: a randomized, controlled, parallel group trial

**DOI:** 10.1186/s12986-024-00799-8

**Published:** 2024-06-10

**Authors:** Piumika Sooriyaarachchi, Ranil Jayawardena, Toby Pavey, Neil A. King

**Affiliations:** 1 Faculty of Health, School of Exercise and Nutrition Sciences, Queensland University of Technology (QUT), Brisbane, QLD Australia; 2 Health and Wellness Unit, Faculty of Medicine, University of Colombo, Colombo, Sri Lanka; 3 Department of Physiology, Faculty of Medicine, University of Colombo, Colombo, Sri Lanka; 4 Nawaloka Hospital Research and Education Foundation, Nawaloka Hospitals PLC, Colombo, Sri Lanka

**Keywords:** Shift work, Obesity, Weight loss, Meal replacement, Body composition

## Abstract

**Background:**

Shift work has been identified as a risk factor for several chronic health conditions including obesity. This study evaluated the impact of a low-calorie meal replacement (MR) as a dinner substitute on body composition and metabolic parameters in shift workers with overweight and obesity.

**Methods:**

An 8-week parallel, randomized controlled trial was conducted on overweight and obese shift workers in a large hospital. An intervention group (IG) (*n* = 25) was provided with a low-calorie MR shake (∼200 kcal) as a replacement for dinner, every day for 8 weeks, while the control group (CG) (*n* = 25) continued their habitual diet. Anthropometric measurements, body composition, biochemical, and lifestyle data were assessed at the first and last visits. Analyses were done per protocol (PP) and by intention to treat (ITT).

**Results:**

Over the study duration, both groups displayed moderate changes in anthropometric measurements and body composition, although these were not statistically significant according to the PP analysis. In the ITT analysis, apart from the hip circumference (HC), all other anthropometric parameters demonstrated significant group and time interactions, suggesting the advantageous effects of the meal replacement over the study period (*P* < 0.05). HDL and VLDL cholesterol measures showed significant main effects, influenced by both group (*P* = 0.031) and time (*P* = 0.050) respectively. The most pronounced dietary shift in the IG was a reduction in carbohydrate consumption and an increase in protein intake. Throughout the study, the meal replacement was well-tolerated, with no adverse events reported.

**Conclusions:**

The meal replacement dietary intervention appears to offer beneficial health effects over time. Extended research is crucial to understand the broader implications of meal replacements across diverse populations.

**Trial registration:**

Australian New Zealand Clinical Trials Registry (ANZCTR): ACTRN12622000231741. Registered on 09 February 2022. https://www.anzctr.org.au/ACTRN12622000231741.aspx.

**Supplementary Information:**

The online version contains supplementary material available at 10.1186/s12986-024-00799-8.

## Introduction

Shift workers are associated with a higher incidence and risk of chronic diseases, including overweight and obesity, metabolic syndrome, cardiovascular diseases (CVDs), type II diabetes, and several types of cancers [1, 2]. Overweight and obesity are significant risk factors for and contributors to increased mortality and comorbidities [3], and several studies show that shift workers are more likely to be overweight or obese than day workers. Obesity has emerged as a significant and persistent issue over several years, exerting an increasing burden on healthcare resources [4].

Overweight and obesity results from complex interactions between environmental and biological factors, with environmental changes contributing to its rapid increase [5]. These factors can be broadly categorized into endogenous (genetic, epigenetic, maternal, hormonal) and exogenous (obesogenic environment, lifestyle, medications) causes [6, 7]. Several factors have been proposed as potential mediators including unhealthy dietary habits, low recreational physical activity, sleep deprivation, increases in alcohol consumption, and disruption of the circadian rhythm [2]. Obesity, characterized by excess fat storage due to an energy imbalance [8], can be addressed through reducing energy intake, increasing expenditure, or a combination of both [9]. Calorie-controlled meal replacements, particularly high-protein variants, offer a safe and effective weight management solution, preserving lean body mass and aiding diet adherence due to their convenience [10, 11].

Shift employment has been linked to obesity via several mechanisms, including circadian disturbance and stress caused by the change in hormonal and metabolic functions [12]. “Circadian disruption” refers to a significant interruption of the internal temporal order of physiological and behavioural circadian rhythms. The principal circadian clock is in the suprachiasmatic nuclei of the hypothalamus (SCN). Circadian clocks have an impact on almost every area of physiology and behaviour, including sleep-wake cycles, cardiovascular activity, endocrine system, body temperature, renal activity, gastrointestinal tract physiology, and hepatic metabolism [13].

Shift work can significantly disrupt sleep patterns and appetite regulation by affecting circadian rhythms and physiological processes [14]. The body’s internal clock regulates hunger and satiety signals, leading to irregular meal patterns and disturbances in satiety cues. Sleep deprivation can further disrupt appetite-controlling hormones like leptin and ghrelin, increasing hunger and food cravings, especially for high-calorie foods [15, 16]. Shift work can also cause mental stress and exhaustion, which can affect eating habits and food preferences. Shift workers may experience an increase in stress-related eating, emotional eating, and reliance on convenience foods [17]. These factors collectively impact dietary patterns and energy balance, potentially contributing to obesity development and progression when combined with disturbed circadian rhythms and altered lifestyle habits [18].

In the weight management of night shift workers meal timing and meal composition are essential for addressing the circadian rhythm of the digestive and metabolic processes as well as any acute physiological effects [19]. Due to the significant impact of meal timing and composition on wakefulness and productivity at work, careful consideration should be given to these factors [20]. In addition, the dietary intervention should be feasible and practical, so that it won’t interfere with their work routine. Therefore, it could be hypothesized that a low-calorie MR intervention would be effective in reducing the body mass of obese shift workers. Thus, the objective of this study was to evaluate the impact of a low-calorie meal replacement (MR) intervention on body mass reduction in shift workers with overweight or obesity.

## Methodology

### Study setting and design

This randomized controlled clinical trial was conducted at the Nawaloka Hospitals PLC, Colombo, Sri Lanka for 8 weeks, evaluating the effect of a low-calorie MR for dinner on shift workers with overweight or obesity. The study included two parallel groups (interventional group/IG [low-calorie MR] and control group/CG [habitual dinner]). Institutional approval was obtained from Nawaloka Hospitals Research and Education Foundation. Ethics was approved by the Queensland University of Technology (QUT) Human Research Ethics Committee (UHREC approval no: 4878) and subsequently registered at the Australian New Zealand Clinical Trials Registry (ACTRN12622000231741). Reporting of the present study is done according to the CONSORT statement (Consolidated Standards for Reporting Trials) (Supplementary File 1). A detailed description of the study protocol is described elsewhere [21].

### Study population and sampling

A sample of 50 shift workers with overweight or obesity was recruited for the study after screening for eligibility criteria. Inclusion criteria included: (a) aged 18–65 years; (b) BMI ≥ 25 kg/m^2^; (c) have engaged in shift work for the last year (d) working at least 3-night shifts/week; and, (e) not having any allergies to any of the known food ingredients, especially for milk and soya. Exclusion criteria were (a) pregnant or lactating women (b) current use of a weight loss medicine/ dietary modification or participating in regular physical activity sessions, (c) having chronic diseases or other untreated illnesses requiring treatment (d) history of any medical surgeries in the past 6 months.

Based on an estimation of the sample size, 50 individuals were needed to determine a 5% reduction in body mass in the IG compared to CG with an 80% power, a 95% confidence interval, and a 30% drop-out rate. Hence, a total of 50 shift workers with overweight or obesity were recruited. Participants were informed about the study, its duration, and participant responsibilities, and recruited only after obtaining informed written consent. Participants were randomly and equally assigned into 2 groups (*n* = 25 each), the MR group (intervention group) and the routine diet group (control group) using the simple random sampling technique. A computer-generated random number sequence was used for randomization. Eligibility assessment and enrolment were done by one independent investigator, while another investigator was involved in randomization.

### The intervention, follow-up, and outcomes

The IG was instructed to consume one serving of the liquid MR (Astron Limited) by adding 4 scoops of MR powder (∼50 g) to 250 mL of water, to replace the habitual dinner meal. Participants were given a shaker bottle to prepare the MR and they were advised to add more water if necessary. The MR contained 20.0 g of protein, 4.5 g of fat, 18.2 g of carbohydrate, and 3.6 g of dietary fibre, and around 200 kcal. The CG was advised to continue with their habitual dinner. All participants were given general dietary and lifestyle advice and asked to continue their usual activities. They were advised to maintain their regular level of physical activity throughout the intervention period, without receiving additional exercise recommendations. Additionally, both groups received general dietary and lifestyle advice through the distribution of leaflets. These leaflets contained basic dietary guidelines based on the Sri Lankan Food-Based Dietary Guidelines, covering topics such as balanced nutrition, portion sizes, and healthy eating habits. The study was conducted for 8 weeks, and the evaluations were done as follows; screening (visit 0), 4 weeks (visit 1), and 8 weeks (visit 2). After each visit, the IG received free MR to last them until the next visit.

A detailed description of the outcomes assessed at each visit is described elsewhere [21]. The primary outcome was the change in body mass from baseline (Seca 874 digital scale, Germany). The secondary outcomes assessed were the changes in the following variables from baseline; glycemic control measures (fasting blood glucose, HbA1c), change in lipid profile [Total cholesterol, Low-Density Lipoprotein (LDL) cholesterol, High Density Lipoprotein (HDL) cholesterol, Very Low-Density Lipoprotein (VLDL) cholesterol, non HDL cholesterol, and triglycerides], change in other anthropometric parameters such as waist circumference (WC), hip circumference (HC), mid upper arm circumference (MUAC) (Seca 201, Germany), and change in systolic and diastolic blood pressure. The change in body composition was assessed by bio-electrical impedance analysis (Bodystat 1500, Bodystat Ltd, Isle of Man, British Isles). Blood pressure was recorded using a digital blood pressure monitor (Omron Healthcare, Singapore). Fat-free mass was calculated using the sex-specific equations developed based on BIA measurements applicable to Asian Indian populations [22].

A venous blood sample of 10–12 mL was collected from each participant after overnight fasting. Serum glucose concentration, plasma total cholesterol, triglycerides, HDL-cholesterol.

will be determined using a Cobas c501 auto analyzer using an electrochemiluminescent immunoassay (ECLIA, Roche Diagnostics). LDL cholesterol will be determined using the Friedewald formula. HbA1c will be evaluated by ion-exchange high-performance liquid chromatography.

A culturally validated food frequency questionnaire FFQ was used to obtain the participants’ habitual intake of calories, macronutrients, and micronutrients [23]. It was administered prior to their enrolment to determine their dietary intake in the month preceding recruitment and again during the final month of the clinical trial to capture their dietary intake during that period. Physical activity was assessed using the translated and validated short version of the International Physical Activity Questionnaire (IPAQ) short form administered by an interviewer at the first and last visits [24]. Additionally, adverse events were noted for safety evaluation.

### Data collection, biochemical analysis, and definitions

Data collection during follow-up visits was carried out by a team of trained research assistants. All anthropometric measurements (height, weight, BMI, WC, and HC) were made by using standard calibrated equipment and following WHO guidelines. Details of anthropometric, clinical, and biochemical measurements have been described in detail elsewhere [21].

### Statistical analysis

Two populations were used in the analyses. The intention-to-treat (ITT) population (*n* = 50) included all subjects who were randomized, while the per-protocol (PP) population (*n* = 39) included all subjects who completed the 8-week intervention (i.e., study completers). All analyses were performed using SPSS version 23 software (SPSS Inc., Chicago, IL, USA), and a *P*-value of < 0.05 was considered statistically significant. All the variables were analysed qualitatively and were expressed as a percentage (%) and numbers (n) The Shapiro-Wilk test was used for all continuous variables to evaluate the normality assumption. To compare the 2 groups for the variables at baseline, independent samples test, Mann-Whitney U test, chi-square or Fisher exact test was used. A two-way analysis of variance (ANOVA), with the treatment group (control vs. intervention) as the between-subjects independent variable and time (baseline and 8 weeks) as the within-subjects independent variable, was used.

ITT analyses were performed on randomized participants with all available data in mixed models as recommended by White and colleagues [25]. The mixed-model analysis allowed for the inclusion of all available data with missing values assumed missing at random. The model had an unstructured covariance matrix to estimate both within and between effects. Group, Time, and Group-Time interactions were included as fixed components, and the restricted maximum likelihood method was used for estimation.

## Results

**Fig. 1:**
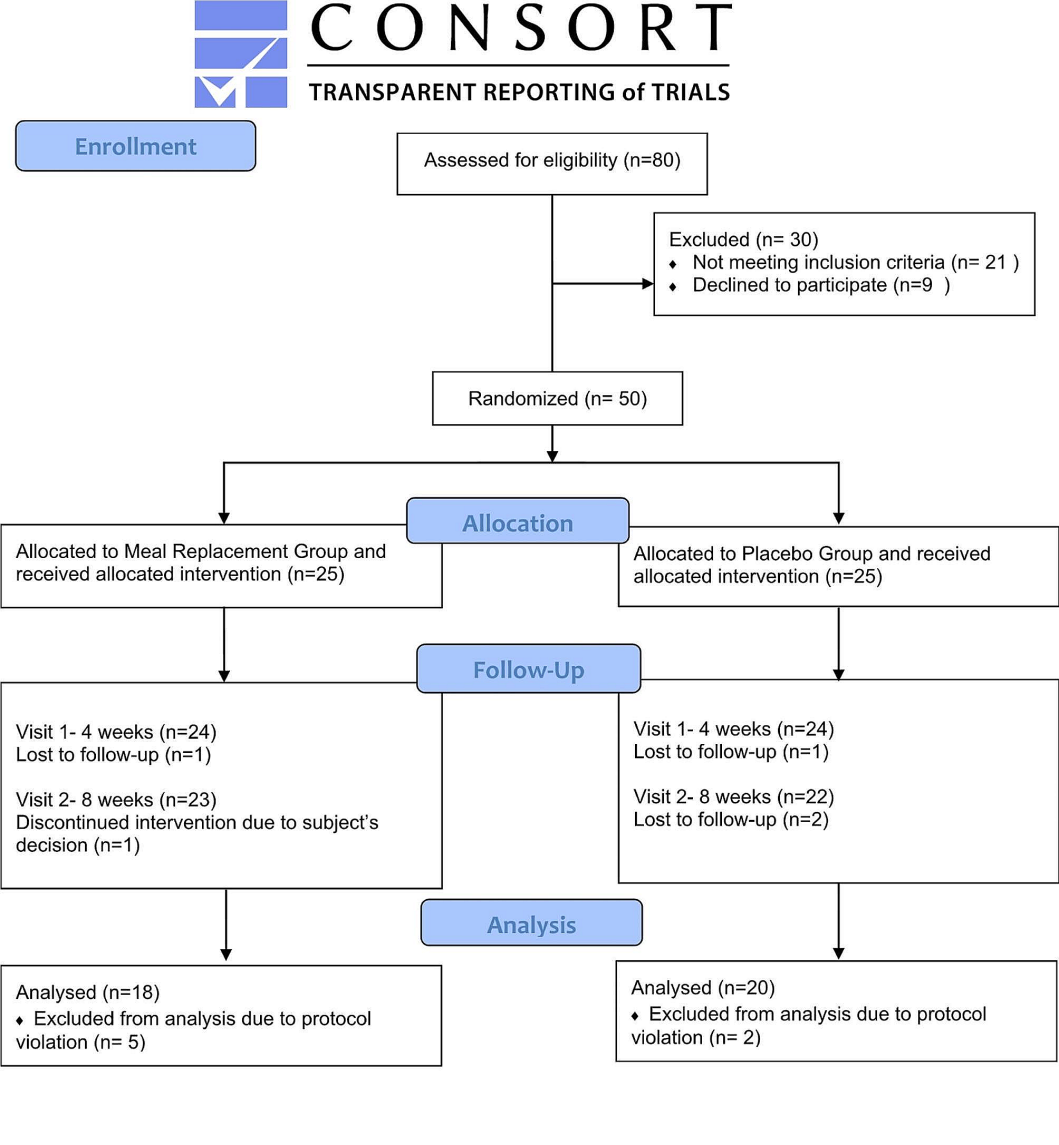
Flow diagram for study selection

Fifty participants (11 males and 39 females), with a mean age (± SD) of 36.02 ± 11.57 years were recruited and randomized to CG and IG. Participant completing 4 and 8-weeks follow-up was 48 and 38 respectively. The reasons for incomplete data were as follows: lost to follow-up (IG = 1, CG = 3), protocol violation (IG = 5, CG = 2), and subject’s decision (IG = 1 and CG = 0). Measurements were obtained from 78% of the sample at an 8-week follow-up (*n* = 39). There was no difference in retention between the IG and CG (χ^2^ = 0.44; *P* = 0.51). A flowchart of the study’s design and the participant dropout rate is shown in Fig. 1. The baseline characteristics of all participants including sociodemographic, clinical, anthropometric, biochemical, and body composition parameters are shown in Table 1. Only HDL cholesterol level was significantly higher in the CG than the IG (*P* = 0.015), and no significant differences were observed for any other measured variables between the IG and CG groups at baseline (*P* > 0.05).

**Table 1:** Baseline characteristics for all randomized subjects

	Control Group (*n* = 25)	Intervention Group (*n* = 25)	*P*-value
Age (years), mean (± SD)	36.8 ± 12.8	35.3 ± 10.4	0.985
**Anthropometric parameters, mean (± SD)**			
Height (cm)	158.4 ± 8.4	160.0 ± 8.1	0.438
Weight (kg)	77.5 ± 15.6	80.2 ± 15.5	0.587
BMI (kg/m^2^)	30.7 ± 3.8	31.2 ± 4.4	0.884
WC (cm)	96.8 ± 12.0	99.9 ± 11.7	0.388
HC (cm)	105.3 ± 8.3	105.7 ± 8.6	0.900
WHR	0.92 ± 0.07	0.95 ± 0.07	0.455
MUAC (cm)	33.1 ± 3.3	34.7 ± 4.3	0.211
**Blood pressure, mean (± SD)**			
Systolic BP (mmHg)	123.1 ± 17.9	120.6 ± 13.5	0.719
Diastolic BP (mmHg)	76.6 ± 9.6	75.2 ± 6.4	0.854
**Body composition, mean (± SD)**			
BF%	42.6 ± 6.4	43.1 ± 6.9	0.930
**Biochemical parameters, mean (± SD)**			
Fasting Plasma Glucose (mg/dL)	96.7 ± 13.9	93.3 ± 10.0	0.406
HbA1c (%)	5.9 ± 0.8	5.7 ± 0.5	0.740
Total cholesterol (mg/dL)	197.5 ± 33.3	187.6 ± 32.8	0.352
LDL cholesterol (mg/dL)	122.6 ± 30.8	120.6 ± 29.0	0.727
HDL cholesterol (mg/dL)	50.2 ± 11.4	42.8 ± 8.1	**0.015**
Triglycerides (mg/dL)	123.0 ± 43.8	120.1 ± 60.9	0.377
VLDL (mg/dL)	24.2 ± 8.7	23.6 ± 12.3	0.361
c/HDL	4.1 ± 1.2	4.5 ± 1.1	0.200
Non-HDL (mg/dL)	147.3 ± 33.3	144.8 ± 32.1	0.621
**Physical activity levels, n (%)**			
Low	14 (56.0%)	10 (40.0%)	0.433
Moderate	8 (32.0%)	13 (52.0%)	
High	3 (12.0%)	2 (8.0%)	
**Dietary intake, mean (± SD)**			
Total energy intake (kcal/day)	1796.5 ± 356.4	1948.5 ± 353.2	0.110
Carbohydrate (g/day)	308.5 ± 53.1	335.8 ± 61.8	0.103
Protein (g/day)	50.6 ± 13.0	55.2 ± 10.6	0.085
Fat (g/day)	44.3 ± 15.9	48.3 ± 13.1	0.197
Dietary fibre (g/day)	17.4 ± 3.5	19.0 ± 3.5	0.137

BMI-Body Mass Index; WC-Waist Circumference; Hip Circumference; WHR-Waist-Hip ratio; MUAC-Mid Upper Arm Circumference; BP- Blood Pressure; BF%-Body Fat Percentage; HDL-High Density Lipoprotein; LDL-Low Density Lipoprotein; VLDL-Very Low-Density Lipoprotein; c/HDL-; Cholesterol to HDL Cholesterol Ratio; SD-Standard Deviation; Bold *P*-values denote statistical significance at the *P* < 0.05 level

### Change in anthropometric and body composition parameters

Changes in anthropometric outcomes over the 8-week intervention period in the PP population are presented in Table 2. Over the span of 8 weeks, the IG exhibited slight reductions in weight, BMI, WC, HC, WHR, and MUAC, closely aligning with the changes observed in the CG. Specifically, the weight in the IG decreased from 78.6 ± 15.0 kg to 76.4 ± 14.8 kg, and BMI from 30.9 ± 4.1 kg/m^2^ to 30.0 ± 4.1 kg/m^2^. Similarly, reductions were noticed in other parameters, such as WC, HC, and MUAC. Nevertheless, the 2-way ANOVA results revealed no statistically significant differences based on group (CG vs. IG), time (baseline vs. 8 weeks), or their interaction across all the anthropometric measures evaluated.

**Table 2:** Changes in anthropometric parameters

Measures	Mean (± SD)				*P*-value			
	Visit 0 (Baseline)		Visit 2 (8 weeks)		Group	Time	Group × Time	
	CG (*n* = 20)	IG (*n* = 18)	CG (*n* = 20)	IG (*n* = 18)				
Weight (kg)	74.7 ± 10.6	78.6 ± 15.0	74.4 ± 10.4	76.4 ± 14.8	0.323	0.676	0.742	
BMI (kg/m^2^)	30.1 ± 3.4	30.9 ± 4.1	30.0 ± 3.4	30.0 ± 4.1	0.647	0.576	0.657	
WC (cm)	94.8 ± 10.1	98.7 ± 11.4	94.1 ± 10.6	94.0 ± 10.8	0.433	0.269	0.420	
HC (cm)	103.8 ± 7.0	104.7 ± 8.1	102.1 ± 6.2	102.9 ± 7.2	0.590	0.290	0.970	
WHR	0.91 ± 0.06	0.94 ± 0.07	0.92 ± 0.08	0.91 ± 0.08	0.510	0.519	0.267	
MUAC (cm)	32.6 ± 3.1	34.0 ± 3.9	31.7 ± 3.2	32.2 ± 3.7	0.254	0.091	0.581	

CG- Control Group; IG- Intervention Group; BMI-Body Mass Index; WC-Waist Circumference; Hip Circumference; WHR-Waist-Hip ratio; MUAC-Mid Upper Arm Circumference; Bold *P*-values denote statistical significance at the *P* < 0.05 level

Considering the changes in body composition (Table 2), the body fat percentage (BF%) for IG reduced slightly from 42.3 ± 5.9% at baseline to 41.4 ± 6.0% at 8 weeks, while the CG maintained almost the same BF% from the baseline to the 8-week mark. Similarly, fat mass in the IG observed a marginal decline, and the fat-free mass demonstrated a minor increase in percentage by the end of the 8 weeks. However, the 2-way ANOVA outcomes indicated no statistically significant differences regarding the group, time, or the interaction between group and time across all the measured body composition parameters.

### Changes in clinical and biochemical parameters

Changes in clinical and biochemical parameters are summarized in Table 3. Both the CG and the IG displayed relatively stable systolic and diastolic blood pressures across the 8-week duration. Similarly, other measures such as fasting blood glucose, HbA1c, total cholesterol, LDL, and VLDL cholesterol demonstrated minimal variations between the groups and over time. Notably, HDL cholesterol levels differed significantly between the groups, with the IG having notably lower values than the CG, as reflected by the *p*-value of < 0.001. The TC/HDL-C ratio also exhibited a statistically significant difference between the two groups (*P* = 0.003). However, for most measures, the 2-way ANOVA results suggested no significant discrepancies attributable to group, time, or their interaction,

**Table 3:** Changes in body composition, blood pressure, and biochemical parameters

	Mean (± SD)				*P*-value		
Measures	Visit 0 (Baseline)		Visit 2 (8 weeks)		Group	Time	Group × Time
	CG (*n* = 20)	IG (*n* = 18)	CG (*n* = 20)	IG (*n* = 18)			
**Body composition**							
BF%	42.3 ± 7.0	42.3 ± 5.9	42.1 ± 6.8	41.4 ± 6.0	0.807	0.707	0.807
Fat mass (kg)	31.8 ± 8.0	33.3 ± 8.5	31.5 ± 7.7	31.7 ± 9.0	0.650	0.635	0.748
Fat-free mass (kg)	42.9 ± 7.1	45.29 ± 9.5	42.9 ± 7.1	44.6 ± 8.8	0.276	0.863	0.850
Fat-free mass %	57.7 ± 7.0	57.7 ± 5.9	57.9 ± 6.8	58.7 ± 6.0	0.807	0.708	0.807
**Blood pressure**							
Systolic BP (mmHg)	125.8 ± 18.1	123.2 ± 13.0	125.9 ± 18.8	122.3 ± 11.1	0.396	0.905	0.889
Diastolic BP (mmHg)	76.9 ± 9.9	76.1 ± 6.9	74.1 ± 9.5	76.6 ± 10.2	0.677	0.606	0.446
**Biochemical**							
Fasting blood glucose (mg/dL)	95.0 ± 14.3	93.1 ± 10.0	92.9 ± 11.6	92.9 ± 5.4	0.715	0.668	0.716
HbA1c (%)	5.7 ± 0.7	5.8 ± 0.5	5.7 ± 0.7	5.7 ± 0.5	0.898	0.462	0.761
Total cholesterol (mg/dL)	195.7 ± 35.1	186.3 ± 28.5	193.6 ± 32.5	185.1 ± 27.2	0.230	0.828	0.952
LDL cholesterol (mg/dL)	119.8 ± 32.7	118.6 ± 27.3	118.7 ± 32.7	122.4 ± 28.6	0.855	0.855	0.733
HDL cholesterol (mg/dL)	52.7 ± 10.6	40.9 ± 6.7	52.4 ± 8.4	42.8 ± 7.6	**< 0.001**	0.700	0.611
Triglycerides (mg/dL)	115.9 ± 44.7	133.1 ± 67.2	113.3 ± 48.0	99.4 ± 27.9	0.885	0.121	0.182
VLDL-C (mg/dL)	22.8 ± 8.9	26.3 ± 13.5	22.4 ± 9.5	19.4 ± 5.6	0.892	0.121	0.167
TC/HDL-C	3.9 ± 1.0	4.7 ± 1.1	3.8 ± 1.0	4.5 ± 1.2	**0.003**	0.670	0.805
Non-HDL-C (mg/dL)	143.0 ± 34.6	145.4 ± 30.1	141.2 ± 33.8	142.3 ± 31.5	0.819	0.757	0.939

CG-Control Group; IG-Intervention Group; BF%-Body Fat Percentage; BP-Blood Pressure, FBS- Fasting Plasma Glucose; LDL-Low Density Lipoprotein; HDL-High Density Lipoprotein; VLDL-Very Low-Density Lipoprotein; TC/HDL-Cholesterol to HDL Cholesterol Ratio; SD-Standard Deviation; Bold *P*-values denote statistical significance at the *P* < 0.05 level

### Change in diet and physical activity

While both groups maintained relatively similar total energy intakes, there were observable variations in specific macronutrient consumption (Table 4). The intake of carbohydrates in the IG slightly reduced over time, a change statistically significant with a *p*-value of 0.041. Also, the IG showed a notable increase in protein consumption compared to the CG, as indicated by a significant *p*-value of 0.004. Fat intake appeared to decrease slightly in the IG, while it increased marginally for the CG; however, these changes weren’t statistically significant. The consumption of dietary fibre was relatively stable across groups and over time. Lastly, the physical activity levels, measured in MET minutes per week, remained consistent for both groups over the duration of the study.

**Table 4:** Changes in diet and physical activity

Measures	Mean (± SD)				*P*-value			
	Visit 0 (Baseline)		Visit 2 (8 weeks)		Group	Time		Group × Time
	CG (*n* = 20)	IG (*n* = 18)	CG (*n* = 20)	IG (*n* = 18)				
Total energy intake (kcal/day)	1722.0 ± 276.0	1932.2 ± 334.5	1711.2 ± 323.6	1720.0 ± 277.8	0.132		0.125	0.166
Carbohydrate (g/day)	295.5 ± 47.0	332.2 ± 57.7	291.2 ± 55.5	285.1 ± 47.7	0.221		**0.041**	0.088
Protein (g/day)	49.3 ± 10.8	55.6 ± 11.4	50.3 ± 15.8	61.2 ± 10.3	**0.004**		0.258	0.444
Fat (g/day)	42.0 ± 11.0	48.6 ± 12.9	44.1 ± 14.3	40.2 ± 12.5	0.661		0.298	0.084
Dietary fibre (g/day)	16.9 ± 3.1	18.5 ± 3.7	16.7 ± 3.5	18.3 ± 4.3	0.067		0.858	0.991
Physical activity (MET minutes/week)	1041.9 ± 950.1	1072.4 ± 1065.0	966.9 ± 886.7	1120.7 ± 1176.2	0.695		0.955	0.793

(CG- Control Group; IG- Intervention Group; SD-Standard Deviation; Bold *P*-values denote statistical significance at the *P* < 0.05 level)

### Mixed-model analysis

Changes in all the measured outcomes over the 8-week intervention period in ITT populations are presented in Table 5. All the anthropometric parameters except the HC showed a significant group × time interactions indicating the benefit of the MR over time (*P* < 0.05). Also, there were main effects of time *P* < 0.001), although no main effects of the group were detected on the anthropometric parameters. Out of the biochemical parameters, HDL and VLDL cholesterol showed the main effect of group (*P* = 0.031) and time (*P* = 0.050) respectively. Neither the interaction nor main effects were significant for other biochemical parameters. For dietary variables, significant treatment effects were found for carbohydrate intake. No treatment or main effects were found for physical activity.

**Table 5:** Fixed effect estimates from the intention-to-treat linear mixed model analysis

Dependant Variables	Group	Visit 0	Visit 2	*P*-value		
				Group	Time	Group × Time
**Anthropometric parameters**						
Weight (kg)	Control	77.5 ± 3.2	77.1 ± 3.1	0.887	**< 0.001**	**< 0.001**
	Intervention	80.2 ± 3.2	77.7 ± 3.1			
BMI (kg/m^2^)	Control	30.7 ± 0.8	30.5 ± 0.8	0.803	**< 0.001**	**< 0.001**
	Intervention	31.2 ± 0.8	30.2 ± 0.8			
WC (cm)	Control	97.0 ± 2.4	96.1 ± 2.4	0.702	**< 0.001**	**< 0.001**
	Intervention	99.9 ± 2.4	94.9 ± 2.4			
HC (cm)	Control	105.3 ± 1.7	103.6 ± 1.6	0.988	**< 0.001**	0.533
	Intervention	105.7 ± 1.7	103.6 ± 1.6			
WHR	Control	0.92 ± 0.01	0.93 ± 0.02	0.654	**< 0.001**	**0.001**
	Intervention	0.95 ± 0.01	0.92 ± 0.02			
MUAC (cm)	Control	33.3 ± 0.8	32.3 ± 0.8	0.685	**< 0.001**	**0.010**
	Intervention	34.7 ± 0.8	32.8 ± 0.8			
**Body composition**						
Hand grip dominant (kg)	Control	20.1 ± 1.7	21.2 ± 1.7	0.458	0.117	0.676
	Intervention	21.3 ± 1.7	23.0 ± 1.8			
Hand grip non-dominant (kg)	Control	18.9 ± 1.7	19.7 ± 1.7	0.666	0.987	0.569
	Intervention	20.8 ± 1.7	20.8 ± 1.7			
BF%	Control	42.6 ± 1.3	42.3 ± 1.4	0.923	**0.005**	0.160
	Intervention	43.1 ± 1.3	42.1 ± 1.4			
**Blood pressure**						
Systolic BP (mmHg)	Control	123.1 ± 3.2	125.9 ± 3.2	0.178	0.676	0.272
	Intervention	120.6 ± 3.2	119.5 ± 3.3			
Diastolic BP (mmHg)	Control	76.6 ± 1.6	74.6 ± 2.1	0.747	0.856	0.326
	Intervention	75.2 ± 1.6	75.5 ± 2.1			
**Biochemical parameters**						
Fasting Plasma Glucose (mg/dL)	Control	96.7 ± 2.6	94.9 ± 2.1	0.437	0.667	0.654
	Intervention	93.3 ± 2.4	92.5 ± 2.1			
HbA1c (%)	Control	5.8 ± 0.1	5.7 ± 0.1	0.658	0.091	0.772
	Intervention	5.7 ± 0.1	5.6 ± 0.1			
Total cholesterol (mg/dL)	Control	197.5 ± 6.6	192.9 ± 6.2	0.371	0.717	0.612
	Intervention	186.3 ± 6.8	184.7 ± 6.5			
LDL cholesterol (mg/dL)	Control	122.6 ± 6.0	119.7 ± 6.1	0.902	0.757	0.499
	Intervention	119.4 ± 6.1	120.9 ± 6.5			
HDL cholesterol (mg/dL)	Control	50.2 ± 1.1	49.8 ± 1.8	**0.031**	0.336	0.300
	Intervention	43.0 ± 2.0	44.0 ± 1.9			
Triglycerides (mg/dL)	Control	123.0 ± 10.7	116.8 ± 8.6	0.206	0.053	0.340
	Intervention	119.4 ± 10.9	100.6 ± 9.2			
VLDL (mg/dL)	Control	24.2 ± 2.2	23.0 ± 1.7	0.187	**0.050**	0.307
	Intervention	23.5 ± 2.2	19.7 ± 1.9			
c/HDL	Control	4.1 ± 0.2	4.1 ± 0.2	0.370	0.246	0.732
	Intervention	4.5 ± 0.2	4.4 ± 0.2			
Non-HDL (mg/dL)	Control	147.3 ± 6.5	143.2 ± 6.6	0.791	0.502	0.805
	Intervention	143.4 ± 6.7	140.6 ± 6.9			
**Dietary intake**						
Total energy intake (kcal/day)	Control	1816.0 ± 73.6	1716.5 ± 61.8	0.860	< 0.001	0.113
	Intervention	1948.5 ± 71.6	1700.7 ± 63.8			
Carbohydrate (g/day)	Control	309.3 ± 12.0	295.5 ± 11.0	0.260	**< 0.001**	**0.013**
	Intervention	335.8 ± 11.6	277.5 ± 11.4			
Protein (g/day)	Control	51.6 ± 2.5	49.2 ± 2.8	**0.010**	0.079	0.063
	Intervention	55.2 ± 2.4	60.0 ± 2.9			
Fat (g/day)	Control	45.6 ± 3.0	43.2 ± 2.9	0.588	**0.004**	0.152
	Intervention	48.3 ± 3.0	41.0 ± 3.0			
Dietary fibre (g/day)	Control	17.4 ± 0.7	16.6 ± 0.8	0.149	0.350	0.985
	Intervention	19.0 ± 0.7	18.3 ± 0.8			
Physical activity (MET mins/week)	Control	1070.6 ± 219.7	959.1 ± 207.9	0.445	0.757	0.969
	Intervention	1286.2 ± 219.7	1191.1 ± 217.6			

BMI-Body Mass Index; WC-Waist Circumference; Hip Circumference; WHR-Waist-Hip ratio; MUAC-Mid Upper Arm Circumference; BF%-Body Fat Percentage; BP-Blood Pressure; HDL-High Density Lipoprotein; LDL-Low Density Lipoprotein; VLDL-Very Low-Density Lipoprotein; c/HDL-; Cholesterol to HDL Cholesterol Ratio; SD-Standard Deviation; Bold *P*-Values denote statistical significance at the *P* < 0.05 level

### Adverse effects and safety

There were no adverse effects reported and none of the participants were hospitalized due to adverse effects during the 8 week follow up period. None of the participants experienced any form of hypersensitivity during the study (immediate and/or delayed).

## Discussion

In this research, we examined the effectiveness of a low-calorie dinner meal replacement for shift workers struggling with overweight or obesity over an 8-week duration. Although several workplace-based weight loss programs have been carried out among obese night shift workers [26, 27], interventions solely focused on diet modification are very limited. To the best of our knowledge, this is the first MR trial to target obese shift workers, and it significantly contributes to the field of workplace-based interventions by demonstrating positive health effects in this vulnerable population.

Throughout the 8-week duration, noticeable variations appeared between the CG and those receiving the meal replacement in terms of weight, BMI, and waist circumference during both visits. However, these differences weren’t always statistically significant. While the intervention group (IG) showed slight reductions in body fat percentage and fat mass, these changes did not reach statistical significance. Both groups displayed stable clinical and biochemical parameters, except for a significant difference in HDL cholesterol levels and the TC/HDL-C ratio. The rise in HDL cholesterol in the intervention group could be linked to the potential weight loss induced by the meal replacement. Weight loss is recognized to have favourable effects on lipid profiles, including elevating HDL cholesterol levels. Higher HDL-C levels are generally associated with reduced cardiovascular disease risk [28], attributed to its role in promoting reverse cholesterol transport and exhibiting anti-inflammatory properties [29, 30]. Additionally, lowering VLDL-C levels can reduce the production of LDL-C, aiding in managing elevated triglyceride levels, which are an independent CVD risk factor [31, 32]. These mechanisms potentially explain the increase in HDL cholesterol levels in the intervention group.

The mixed-model analysis within the ITT population further supplemented these findings, revealing that the meal replacement seems to offer significant benefits over time when assessing anthropometric parameters, as indicated by the significant group × time interactions. Except for hip circumference (HC), all anthropometric measurements reflected this trend, underscoring the potential efficacy of the MR intervention. Additionally, while time exerted a pronounced effect on these measures (*P* < 0.001), the group itself did not independently influence the anthropometric outcomes. Considering biochemical parameters, two markers, HDL and VLDL cholesterol, stood out. The former exhibited significant variations based on the group (*P* = 0.031) and the latter based on time (*P* = 0.050). It is crucial to note that apart from these two markers, the other biochemical parameters did not exhibit significant interaction or main effects.

Additionally, the body mass reduction reported in the present study did not reach clinical significance, typically set at a 5-10% decrease [33]. Given the short duration of this research (8 weeks), it is expected that future studies with longer durations will be able to achieve clinically meaningful weight loss from baseline which is thought to be at least 5%. However, even modest amounts of weight loss have demonstrated multiple metabolic and cardiovascular risk factor benefits [34], such as improvement in systolic and diastolic blood pressure and HDL cholesterol [35]. This is supported by the fact that in the current trial, the overall body mass loss of nearly 3% in the MR group at the end of 8 weeks was accompanied by significant increase in HDL cholesterol and a reduction in VLDL cholesterol.

Moreover, a reduction in BF% was observed in the IG at the end of 8 weeks. These results are consistent with those of other low-calorie MR intervention trials where significant drops in BF% have been noted [36, 37]. A reduction in fat mass has substantial clinical relevance, as it can improve insulin sensitivity, decrease inflammation, optimize lipid profiles, reduce cardiovascular risk, and enhance overall metabolic function [38, 39]. These effects contribute to a decreased risk of chronic diseases such as type 2 diabetes, CVD, and metabolic syndrome, ultimately promoting better overall health and well-being [40]. A primary objective of modern obesity treatments is to maximize fat loss while maintaining lean tissue mass and function. It is essential for favourable metabolic benefits, weight reduction maintenance, and sarcopenic obesity caused by the loss of muscle mass [41]. The assessment of the clinical utility of weight-reduction programmes should focus on body composition measures such as free fat mass, where the ratio of free-fat mass loss to weight loss may serve as a biomarker of clinical efficacy [42].

Significantly, the IG demonstrated a marked decrease in carbohydrate consumption and an increase in protein intake compared to the CG. This could be attributed to the intake of the meal replacement (MR) which had reduced carbohydrates and elevated protein levels compared to their typical dinner meal, which is generally rich in fats and carbohydrates. It is well known that excess carbohydrate and fat intake places a large metabolic load on the body which eventually leads to obesity and metabolic disarrangement [43].

However, this study has several limitations. Firstly, the short 8-week follow-up period restricts the assessment of long-term program effectiveness. Secondly, the relatively small sample size in comparison to other weight loss trials hinders the generalizability of findings. Therefore, future studies with larger samples and extended follow-up periods are needed to establish a stronger evidence base for policy reform. The use of Food Frequency Questionnaires (FFQs) presents limitations compared to dietary recalls and food diaries. Participants may struggle to accurately recall and report food consumption frequency and portion sizes, leading to potential recall bias. FFQs utilize predefined portion sizes, overlooking individual variations and impacting dietary intake accuracy [44]. Moreover, they rely on predetermined food lists, potentially missing specific foods consumed and providing only an overall estimation of dietary intake, which may overlook day-to-day variations. Therefore, incorporating multiple dietary assessment methods could enhance the reliability of results and provide a more comprehensive understanding of the program’s impact. Moreover, ur study did not involve a formal assessment of sleep quality and duration among participants. Despite this limitation, future studies could explore the potential interplay between sleep patterns and dietary interventions to provide a more comprehensive understanding of factors influencing metabolic health in this population.

This study has several strengths that contribute to the credibility of its findings. The main strength of this study was the randomized controlled study design and the inclusion of shift workers with overweight or obesity and not following any other weight loss treatments. The study also assessed several additional health outcomes including clinical, biochemical, body composition and anthropometric changes while adhering to a protocol. The trial included face-to-face collection of data by trained research assistants and all anthropometric measurements were taken using standard equipment and techniques. The study also measured participants dietary intake, physical activity level and the sleep quality using validated questionnaires. The study was deemed to be practical because the retention rate was high, resulting in the retention of 78% of the initial sample at the end of two months, and no adverse events were noted. Also, from a safety perspective, the meal replacement regimen was well-tolerated among the participants. There were no instances of adverse reactions, hospitalizations, or hypersensitivity responses documented throughout the study period.

In conclusion, the low-calorie MR intervention demonstrated a moderate reduction in body mass, reduction in WC, BMI, WHR and BF%. Additionally, the intervention showed a beneficial reduction in VLDL cholesterol and improvements HDL cholesterol in shift workers with overweight or obesity. To examine the long-term consequences of consuming the MR, further follow-up studies with larger sample sizes are recommended.

### Electronic supplementary material

Below is the link to the electronic supplementary material.

Supplementary Material 1. CONSORT checklist.

## Data Availability

The datasets used and/or analysed during the current study are available from the corresponding author on reasonable request.

## References

[1] Wang X, Armstrong M, Cairns B, Key T, Travis R (2011). Shift work and chronic disease: the epidemiological evidence. Occup Med.

[2] Knutsson A (2003). Health disorders of shift workers. Occup Med.

[3] Ekmekcioglu C, Touitou Y (2011). Chronobiological aspects of food intake and metabolism and their relevance on energy balance and weight regulation. Obes Rev.

[4] Zhang Q, Chair SY, Lo SHS, Chau JP-C, Schwade M, Zhao X (2020). Association between shift work and obesity among nurses: a systematic review and meta-analysis. Int J Nurs Stud.

[5] Loos RJF, Yeo GSH (2022). The genetics of obesity: from discovery to biology. Nat Rev Genet.

[6] Hruby A, Hu FB (2015). The epidemiology of obesity: a big picture. PharmacoEconomics.

[7] Saxena I, Suman S, Kaur AP, Mitra P, Sharma P, Kumar M (2021). The multiple causes of obesity. Role of obesity in Human Health.

[8] Faria SL, Faria OP, Menezes CS, de Gouvêa HR, de Almeida Cardeal M (2012). Metabolic profile of clinically severe obese patients. Obes Surg.

[9] Thomas D, Bouchard C, Church T, Slentz C, Kraus W, Redman L (2012). Why do individuals not lose more weight from an exercise intervention at a defined dose? An energy balance analysis. Obes Rev.

[10] Astbury NM, Piernas C, Hartmann-Boyce J, Lapworth S, Aveyard P, Jebb SA (2019). A systematic review and meta‐analysis of the effectiveness of meal replacements for weight loss. Obes Rev.

[11] Mettler S, Mitchell N, Tipton KD (2010). Increased protein intake reduces lean body mass loss during weight loss in athletes. Med Sci Sports Exerc.

[12] Hulsegge G, Proper KI, Loef B, Paagman H, Anema JR, van Mechelen W (2021). The mediating role of lifestyle in the relationship between shift work, obesity and diabetes. Int Arch Occup Environ Health.

[13] Reppert SM, Weaver DR (2002). Coordination of circadian timing in mammals. Nature.

[14] Boege HL, Bhatti MZ, St-Onge M-P (2021). Circadian rhythms and meal timing: impact on energy balance and body weight. Curr Opin Biotechnol.

[15] Damiola F, Le Minh N, Preitner N, Kornmann B, Fleury-Olela F, Schibler U (2000). Restricted feeding uncouples circadian oscillators in peripheral tissues from the central pacemaker in the suprachiasmatic nucleus. Genes Dev.

[16] Taheri S, Lin L, Austin D, Young T, Mignot E (2004). Short sleep duration is associated with reduced leptin, elevated ghrelin, and increased body mass index. PLoS Med.

[17] Canuto R, Garcez A, Spritzer PM, Olinto MTA (2021). Associations of perceived stress and salivary cortisol with the snack and fast-food dietary pattern in women shift workers. Stress.

[18] Nea FM, Kearney J, Livingstone MBE, Pourshahidi LK, Corish CA (2015). Dietary and lifestyle habits and the associated health risks in shift workers. Nutr Res Rev.

[19] Lowden A, Moreno C, Holmbäck U, Lennernäs M, Tucker P. Eating and shift work—effects on habits, metabolism, and performance. Scand J Work Environ Health. 2010:150–62. 10.5271/sjweh.2898 20143038

[20] Phoi YY, Keogh JB (2019). Dietary interventions for night shift workers: a literature review. Nutrients.

[21] Sooriyaarachchi P, Jayawardena R, Pavey T, King N. Meal replacement as a weight loss strategy for night shift workers with obesity: a protocol for a randomized controlled trial. Trials. 2022. 10.1186/s13063-022-06784-x PMC9548175 36209132

[22] Rush E, Chandu V, Plank L (2006). Prediction of fat-free mass by bioimpedance analysis in migrant Asian Indian men and women: a cross validation study. Int J Obes.

[23] Jayawardena R, Swaminathan S, Byrne NM, Soares MJ, Katulanda P, Hills AP (2012). Development of a food frequency questionnaire for Sri Lankan adults. Nutr J.

[24] Craig CL, Marshall AL, Sjöström M, Bauman AE, Booth ML, Ainsworth BE (2003). International physical activity questionnaire: 12-country reliability and validity. Med Sci Sports Exerc.

[25] White IR, Carpenter J, Horton NJ (2012). Including all individuals is not enough: lessons for intention-to-treat analysis. Clin Trails.

[26] Morgan PJ, Collins CE, Plotnikoff RC, Cook AT, Berthon B, Mitchell S (2011). Efficacy of a workplace-based weight loss program for overweight male shift workers: the Workplace POWER (preventing obesity without eating like a rabbit) randomized controlled trial. Prev Med.

[27] Morgan PJ, Collins CE, Plotnikoff RC, Cook AT, Berthon B, Mitchell S et al. The impact of a workplace-based weight loss program on work-related outcomes in overweight male shift workers. J Occup Environ Med. 2012:122–7. 10.1097/JOM.0b013e31824329ab 22269987

[28] Cho YK, Jung CH (2021). HDL-C and Cardiovascular Risk: you don’t need to worry about extremely high HDL-C levels. J Lipid Atheroscler.

[29] Rosenson RS, Brewer HB, Davidson WS, Fayad ZA, Fuster V, Goldstein J (2012). Cholesterol efflux and atheroprotection: advancing the concept of reverse cholesterol transport. Circulation.

[30] Murphy AJ, Woollard KJ, Hoang A, Mukhamedova N, Stirzaker RA, McCormick SP (2008). High-density lipoprotein reduces the human monocyte inflammatory response. Arterioscler Thromb Vasc Biol.

[31] Adiels M, Olofsson S-O, Taskinen M-R, Borén J (2008). Overproduction of very low–density lipoproteins is the hallmark of the dyslipidemia in the metabolic syndrome. Arterioscler Thromb Vasc Biol.

[32] Hokanson JE, Austin MA (1996). Plasma triglyceride level is a risk factor for cardiovascular disease independent of high-density lipoprotein cholesterol level: a metaanalysis of population-based prospective studies. J Cardiovasc Risk.

[33] Jensen MD, Ryan DH, Apovian CM, Ard JD, Comuzzie AG, Donato KA (2014). 2013 AHA/ACC/TOS guideline for the management of overweight and obesity in adults: a report of the American College of Cardiology/American Heart Association Task Force on Practice guidelines and the obesity society. J Am Coll Cardiol.

[34] Magkos F, Fraterrigo G, Yoshino J, Luecking C, Kirbach K, Kelly SC (2016). Effects of moderate and subsequent progressive weight loss on metabolic function and adipose tissue biology in humans with obesity. Cell Metabol.

[35] Ryan DH, Yockey SR (2017). Weight loss and improvement in Comorbidity: differences at 5%, 10%, 15%, and over. Curr Obes Rep.

[36] Basciani S, Costantini D, Contini S, Persichetti A, Watanabe M, Mariani S (2015). Safety and efficacy of a multiphase dietetic protocol with meal replacements including a step with very low calorie diet. Endocrine.

[37] Ard JD, Lewis KH, Rothberg A, Auriemma A, Coburn SL, Cohen SS (2019). Effectiveness of a total meal replacement program (OPTIFAST Program) on weight loss: results from the OPTIWIN Study. Obesity.

[38] Shoelson SE, Herrero L, Naaz A (2007). Obesity, inflammation, and insulin resistance. Gastroenterology.

[39] Klop B, Elte JWF, Castro Cabezas M (2013). Dyslipidemia in obesity: mechanisms and potential targets. Nutrients.

[40] Eckel RH, Grundy SM, Zimmet PZ (2005). The metabolic syndrome. Lancet.

[41] Santarpia L, Contaldo F, Pasanisi F (2013). Body composition changes after weight-loss interventions for overweight and obesity. Clin Nutr.

[42] Heymsfield SB, Gonzalez MC, Shen W, Redman L, Thomas D (2014). Weight loss composition is one-fourth fat‐free mass: a critical review and critique of this widely cited rule. Obes Rev.

[43] Liu S (2002). Intake of refined carbohydrates and whole grain foods in relation to risk of type 2 diabetes mellitus and coronary heart disease. J Am Coll Nutr.

[44] Jayawardena R, Byrne NM, Soares MJ, Katulanda P, Hills AP (2016). Validity of a food frequency questionnaire to assess nutritional intake among Sri Lankan adults. SpringerPlus.

