# Supplementary material for: A low-calorie meal replacement improves body composition and metabolic parameters in shift workers with overweight and obesity: a randomized, controlled, parallel group trial
Source: Nutr Metab (Lond). 2024 Jun 10;21:32. doi: 10.1186/s12986-024-00799-8 (PMC11165784; doi:10.1186/s12986-024-00799-8)
Supplement: Supplementary file 1 — Supplementary Material 1. CONSORT checklist. [file 12986_2024_799_MOESM1_ESM.docx]

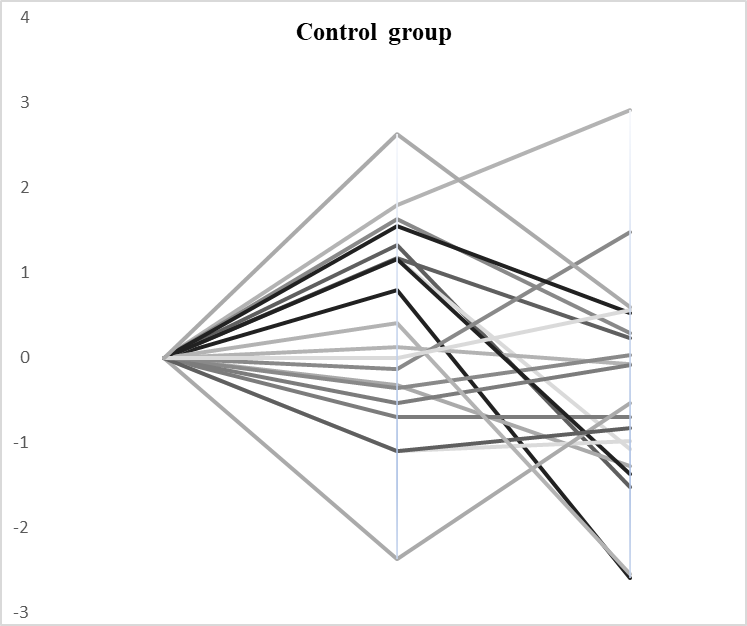


Baseline 4 weeks 8 weeks

Percentage weight change

**Supplementary file 2.** Percentage weight change of the groups at 4 weeks and 8 weeks
